# Association between Mediterranean Diet and Type 2 Diabetes: Multiple Cross-Sectional Analyses

**DOI:** 10.3390/nu15133025

**Published:** 2023-07-03

**Authors:** Adèle Bossel, Gérard Waeber, Antoine Garnier, Pedro Marques-Vidal, Vanessa Kraege

**Affiliations:** 1 Faculty of Biology and Medicine, Lausanne University, UNIL, 1015 Lausanne, Switzerland; 2 Department of Medicine, Internal Medicine, Lausanne University Hospital, 1012 Lausanne, Switzerland; gerard.waeber@chuv.ch (G.W.); pedro-manuel.marques-vidal@chuv.ch (P.M.-V.); vanessa.kraege@chuv.ch (V.K.); 3 Medical Directorate, Lausanne University Hospital, 1012 Lausanne, Switzerland; antoine.garnier@chuv.ch; 4 Innovation and Clinical Research Directorate, Lausanne University Hospital, 1012 Lausanne, Switzerland

**Keywords:** Mediterranean diet, type 2 diabetes, nutrition, non communicable diseases, diet score, adherence

## Abstract

Aim: To assess whether the Mediterranean diet (MD) is associated with lower levels of type 2 diabetes (T2D) in a non-Mediterranean population. Methods: Cross-sectional analysis of follow-ups 1 (FU1, 2009–2012, *n* = 4398, 45.7% men, 57.7 ± 10.5 years), 2 (FU2, 2014–2017, *n* = 3154, 45.0% men, 61.7 ± 9.9 years), and 3 (FU3, 2018–2021, *n* = 2394, 45.2% men, 65.0 ± 9.6 years) of the Colaus|PsyCoLaus study (Lausanne, Switzerland). Two MD scores (Trichopoulou, noted MD1, and Sofi, noted MD2) were calculated using participants’ dietary data. T2D was defined as a fasting plasma glucose ≥7 mmol/L and/or the presence of an antidiabetic drug treatment. Results: Participants with the highest MD adherence had a higher educational level, a lower BMI, were less frequent smokers, presented less frequently with hypertension, and were more frequent alcohol consumers. After multivariable adjustment, no differences were found between participants with and without T2D regarding MD scores: 3.93 ± 0.07 vs. 3.97 ± 0.02; 4.08 ± 0.10 vs. 3.98 ± 0.03, and 3.83 ± 0.11 vs. 3.97 ± 0.03, respectively, for the MD1 score in FU1, FU2, and FU3. In addition, no association was found between adherence to MD and T2D: odds ratio (and 95% confidence interval) for medium and high relative to low adherence to MD1: 0.87 (0.68–1.10) and 0.89 (0.64–1.24) in FU1, 1.04 (0.76–1.42) and 1.07 (0.68–1.67) in FU2, and 0.73 (0.53–1.03) and 0.61 (0.37–1.02) in FU3, respectively. Corresponding results for MD2 were 0.90 (0.70–1.15) and 1.03 (0.69–1.53) in FU1, 1.16 (0.82–1.63) and 1.40 (0.81–2.41) in FU2, and 0.93 (0.65–1.34) and 0.55 (0.28–1.08) in FU3. Conclusion: We found no association between Mediterranean diet adherence and T2D in a non-Mediterranean population.

## 1. Introduction

Type 2 diabetes (T2D) is an epidemic disease in Western countries with a global prevalence expected to increase from 427 million people in 2017 to 592 million in 2035, representing a heavy clinical burden [1].

T2D prevalence depends on several factors such as genetics, age, physical activity (PA), and diet. To delay T2D prevalence, the most effective means of prevention are those aimed at modifying environmental risk factors by reducing obesity and promoting PA [2]. Several diets are known to effectively reduce obesity, such as the Mediterranean diet (MD) [3]. The MD is traditionally practiced in countries around the Mediterranean Sea such as Greece, Turkey, and Lebanon, and is associated with olive cultivation in those areas [4]. It consists of a high consumption of fruits, vegetables, olive oil, legumes, nuts, and fish, a moderate consumption of low-fat dairy products and alcohol, and a low consumption of red meat [5]. Such diets have also been observed beyond the Mediterranean area [6,7,8].

Indeed, many positive effects on health have been associated with the MD, such as cardiovascular risk factors (hypertension, obesity, metabolic syndrome, dyslipidemia), mortality, and neurodegenerative disorders [4,9,10]. Moreover, the MD has a positive climate impact compared to less sustainable food consumption patterns [11].

Many studies have demonstrated an inverse association between the MD and T2D prevalence [12,13]. Moreover, the ability of the MD to reduce T2D prevalence has been shown in many populations [12,13,14,15]. However, one study showed that despite high adherence to the MD being associated with lower insulin and blood glucose levels, it was not associated with a decreased incidence of diabetes [16].

Therefore, this study aims to investigate the association between T2D prevalence and MD adherence in Lausanne, Switzerland. We want to study the effect of a MD in a non-Mediterranean country, where the healthcare system is very developed, the socio-economic status is especially high, and the obesity rate is one of the lowest in Western countries [17]. Our hypothesis is that the higher the adherence to a MD, the lower the prevalence of T2D. Our secondary objective is to investigate subjects’ specific characteristics depending on their MD adherence. Our hypothesis is that high education level, low BMI, no-smoking status, low energy intake, hypertension, and low alcohol consumption tend to be associated with high MD adherence.

## 2. Materials and Methods

### 2.1. Population and Study Design

Data from the CoLaus|PsyCoLaus study were used. The CoLaus|PsyCoLaus is a population-based prospective study assessing the clinical, biological, and genetic determinants of cardiovascular disease in subjects aged 35 to 75 years living in the city of Lausanne, Switzerland. Recruitment began in 2003 and ended in 2006. The first follow-up was performed between 2009 and 2012, the second between 2014 and 2017, and the third between 2018 and 2021. In each survey, participants answered questionnaires, underwent a clinical examination, and blood samples were drawn for analyses. For more details, see www.colaus-psycolaus.ch. As dietary intake was not assessed at baseline, only data from the three follow-ups were used.

### 2.2. Dietary Assessment

Dietary intake was assessed using a self-administered, semi-quantitative food frequency questionnaire (FFQ) which also included portion size. This FFQ has been validated in the Geneva population [18]. Briefly, this FFQ assesses the dietary intake of the 4 previous weeks and consists of 97 different food items that account for more than 90% of the intake of calories, proteins, fat, carbohydrates, alcohol, cholesterol, vitamin D, and retinol, and 85% of fiber, carotene, and iron. For each item, consumption frequencies ranging from “less than once during the last 4 weeks” to “2 or more times per day” were provided, and the participants also indicated the average serving size (smaller, equal, or bigger) compared to a reference size. Each participant brought along their filled-in FFQ, which was checked for completion by trained interviewers on the day of the appointment.

### 2.3. Quantification of the Mediterranean Diet Adherence Scores

As data from three follow-ups were available, two methods were used to compute the MD adherence scores, as performed previously [14,19]. The first method used the MD score as defined by Trichopoulou et al. [14], in which 8 food types are considered. These were legumes, cereals (including bread and potatoes), fruits, vegetables, meat and meat products, milk and dairy products, monounsaturated: saturated fat ratio, and ethanol [14]. Foods considered a priori as beneficial received a score of 1 if their consumption exceeded the median of the sample, and of 0 if below the median. These scores were reversed for foods estimated a priori as detrimental (1 if below the median, 0 if above) [20]. The final score thus ranges from 0 to 9, a score of 0 reflecting low adherence and 9 high adherence. This score was designated as MD1 and was further categorized as low (<4), medium (≥4 and <6), and high (≥6) adherence, as in Trichopoulou et al. [14].

We also computed a MD score as defined by Francesco Sofi et al. [19]. This score includes vegetables, fruit, dairy products, cereals, meat and meat products, fish, alcohol, and olive oil. Standard portions were determined according to the literature and allowed to scale the consumption of the research subjects of the 3 follow-ups. For beneficial foods, high consumption received a score of 2, medium consumption a score of 1, and low consumption one of 0. The opposite is applied to detrimental foods, which received a score of 0 if much consumed, 1 if moderately consumed, and 2 if little consumed [19]. The foods used to compute the Sofi score are indicated in Table S1. This score ranges from 0 to 18; it was designated as MD2 and was further categorized as low (<5), medium (≥5 and <9), and high (≥9) [21].

### 2.4. Diabetes

For the main analyses, T2D was defined as a fasting plasma glucose (FPG) ≥7 mmol/L and/or the presence of an oral antidiabetic and/or insulin treatment. Sensitivity analyses were conducted by defining T2D as glycated hemoglobin ≥6.5% (48 mmol/mol).

### 2.5. Other Covariates

Participants self-filled questionnaires on socio-economic and health data. Educational level was categorized as low (primary), middle (apprenticeship), upper middle (high school), and high (university) for the highest completed level of education. Smoking status was self-reported and categorized as never, former, and current. Alcohol consumption was categorized into none, 1–7 units/week, and >7 units/week (1 unit = 1 glass of wine, 1 can of beer, or 1 shot of spirit). The presence of a diet (low sugar/diabetic, low salt, etc.) and a family history of T2D were also collected.

Body weight and height were measured with participants barefoot and in light indoor clothes. Body weight was measured in kilograms to the nearest 100 g using a Seca^®^ scale (Hamburg, Germany). Height was measured to the nearest 5 mm using a Seca^®^ (Hamburg, Germany) height gauge. Body mass index (BMI) was computed and categorized into normal (BMI < 25 kg/m^2^), overweight (25 ≤ BMI < 30 kg/m^2^), and obese (BMI < 30 kg/m^2^).

Blood pressure (BP) was measured thrice using an Omron^®^ HEM-907 (Matsusaka, Japan) automated oscillometric sphygmomanometer after at least a 10-min rest in a seated position. Hypertension was defined by a systolic BP ≥ 140 mm Hg and/or a diastolic BP ≥ 90 mm Hg and/or the presence of antihypertensive drug treatment.

### 2.6. Laboratory Data

Biological assays were performed by the CHUV Clinical Laboratory on fresh blood samples within 2 h of blood collection. All measurements were performed in a Cobas 8000 (Roche Diagnostics, Basel, Switzerland). FPG was assessed by glucose hexokinase, with maximum inter- and intra-batch CVs of 1.6–0.8%, respectively. In the second and third follow-ups, glycated hemoglobin levels were measured by high-performance liquid chromatography using Bio-Rad, D-10^TM^ system, with a measurement range of 3.8% (18 mmol/mol) to 18.5% (179 mmol/mol).

### 2.7. Inclusion and Exclusion Criteria

Participants were eligible if they participated in the different follow-ups. Within each follow-up, participants were excluded if they (1) missed nutritional data; (2) presented extreme total energy intake values (defined as <500 or >3500 kcal/day for women and <800 or >4000 kcal/day for men) [22]; (3) missed data regarding diabetes status; or (4) missed covariates.

### 2.8. Statistical Analysis

Statistical analyses were performed separately for each follow-up using Stata version 16.1 for Windows (Stata Corp, College Station, TX, USA) and R (www.r-project.org accessed on 9 June 2023). Descriptive results were expressed as the number of participants (percentage) for categorical variables and as average ± standard deviation or median [interquartile range] for continuous variables. Adherence to the Mediterranean diet between subjects with and without T2D was compared using Mediterranean diet scores as continuous variables or categorized as described previously.

Bivariate analyses were performed using chi-square or Fisher’s exact test for categorical variables and Student’s *t*-test, analysis of variance (ANOVA), or Kruskall–Wallis test for continuous variables. Multivariable analysis of categorical variables was performed using logistic regression and the results were expressed as Odds ratio (OR) and 95% confidence interval (CI). Multivariable analysis of continuous variables was performed using ANOVA and results were expressed as multivariable-adjusted mean ± standard error. For multivariable analyses, adjustment was performed on age (continuous), sex, place of birth (Swiss-born, yes or no), educational level (high, medium, low), smoking categories (never, former, current), BMI categories (normal, overweight, obese), hypertension (yes or no), alcohol consumption (yes or no), and total caloric intake (continuous).

We also performed a sensitivity analysis using inverse probability weighting to take into account excluded participants. Briefly, logistic regression was used to estimate the likelihood of being included for each participant using covariates that were significantly different between included and excluded participants [23]. The inverse of predicted probability was then used for the analyses by logistic regression.

Finally, to check if a more specific association exists between low-fat intake and T2D risk reduction, we conducted bi- and multivariable analyses of fat consumption (total, saturated, monounsaturated, polyunsaturated) to compare participants with or without T2D using the same statistical analysis as above.

Statistical significance was assessed for a two-sided test with *p* < 0.05.

## 3. Results

### 3.1. Selection and Characteristics of the Participants

**Figure 1:**
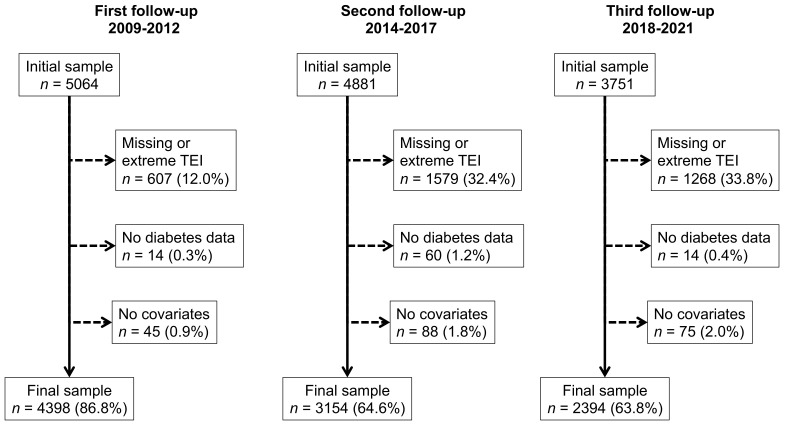
Exclusion criteria and number of participants for each follow-up.

The reasons for exclusion for each follow-up are summarized in Figure 1 and the characteristics of the excluded and included participants are indicated in Table S2. Excluded participants were older, more frequently of a lower educational level, presented more frequently with overweight or obesity, had higher levels of hypertension and diabetes, and lower levels of alcohol consumption.

Participant characteristics for each follow-up according to categories of adherence to MD1 and MD2 are indicated in Tables S3 and S4, respectively. Overall, participants with higher scores had a higher educational level, a lower BMI, were less frequently current smokers, presented less frequently with hypertension, and were more frequently alcohol consumers.

### 3.2. Mediterranean Diet and Diabetes

**Figure 2:**
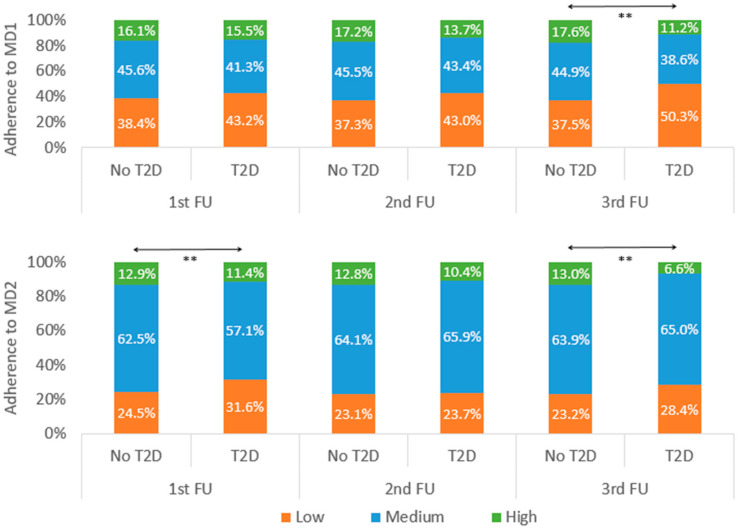
Bivariate analysis of the associations between the different Mediterranean diet scores and type 2 diabetes as defined by fasting plasma glucose ≥ 7 mmol/L, stratified by survey period, CoLaus|PsyCoLaus study, Lausanne, Switzerland. Between-group comparisons performed using chi-square. Value significance ** < 0.01. T2D, type 2 diabetes; FU, follow-up; MD1, Trichopoulou score; MD2, Sofi score.

Bi- and multivariable associations between categories of adherence to MD1 and MD2 and MD1 and MD2 scores and prevalence of T2D for each follow-up are indicated in Figure 2 and Table 1. Overall, there were few statistically significant associations between MDs and prevalent T2D, except for MD2 in the first follow-up and for both MD1 and MD2 in the third follow-up, where participants devoid of T2D had higher scores. Analyses using HbA_1_c to define T2D (Table 2) or using inverse probability weighting to account for excluded participants (Table S5) led to similar findings. Finally, bi- and multivariable comparisons of fat intake between participants with and without T2D showed no significant differences except for polyunsaturated fat, which was consumed more by participants with T2D in bivariate FU1 and FU2; and multivariable FU2 (Table S6).

## 4. Discussion

Contrary to our initial hypothesis, we failed to find an association between the Mediterranean diet and prevalence of T2D in a non-Mediterranean population.

### 4.1. Participants Adhering to the Mediterranean Diet

As expected, participants with high MD adherence had a higher educational level, a lower BMI, were more frequently nonsmokers, and presented less frequently with hypertension. In MD2 only, participants with high MD adherence were more frequently women and less frequently drank alcohol, whereas in MD1, participants with higher adherence were more frequently alcohol consumers and no significant difference in sex could be shown. Our findings are partly in agreement with a Spanish study, which showed that MD adherence was higher in women, older subjects, former smokers, and more physically active participants [24]. They also partly agree with a Greek study, which showed that more educated participants tended to have higher adherence to a MD [25]. Overall, our results indicate that participants adhering to the MD tend to be more health conscious than those who do not.

### 4.2. Mediterranean Diet and Diabetes

The exact mechanisms related to the benefits of a MD on T2D are not exactly known. Possible mechanisms include the lipid reduction effect, the prevention of inflammation and oxidative stress, the restriction of nutrient sensing pathways by limiting certain amino acids, and the induction by gut microbiota of metabolites influencing metabolic health [20]. Indeed, the reduction in T2D prevalence with a MD may partly be explained by the effect of virgin olive oil on insulin resistance [16], and more broadly by that of the entire MD on BMI, lipoprotein metabolism, adiponectin level, and concentration of inflammatory markers [22,23,24].

No association between MD1 and T2D was found in our study. Our finding does not replicate those of studies from Iran [12], Spain [26], and Greece [25], which show an inverse association between a MD and T2D. A likely explanation for our null finding is that the method to compute a MD might be inadequate in non-Mediterranean countries [27], as the types of food consumed as well as their methods of production and preparation are different. Indeed, the use of the population-specific median as a threshold is likely inadequate in populations whose consumption of Mediterranean-type foods is low. For example, a participant living in Switzerland might be given a score of 1 for fruit consumption, whereas they would have been given a score of 0 had they lived in Greece. Hence, due to its definition, it is likely that the MD1 score is overestimated and might not be the best metric to evaluate adherence to a healthy diet in non-Mediterranean countries. Further, the positive effects of a MD are not necessarily applicable to all populations [28] and a diet adapted to the culture, genetics, and environment of each population might be more beneficial [29].

Moreover, in our study, the lack of association between a MD and T2D could not be explained by a counterbalancing the association between fat intake and diabetes risk. The only significant difference was enhanced consumption of polyunsaturated fats in diabetics of the first two follow-ups, as participants’ general practitioners had probably recommended them to change their consumption since their T2D diagnosis [30]. This observation differs from Barnard and Kahleova, who observed a lowering effect on insulin resistance by introducing a low-fat vegan diet [31,32,33]. It may be that these diets differ importantly from the usual calorie intake as they recommend a fat intake of only 10% of total calories, which is half the lower recommendation for lipid intake in Switzerland [34].

To our knowledge, no study has associated Sofi score with T2D. Sofi et al. had shown that a high level of adherence to this score reduced mortality, and cardiovascular and neoplastic risk [19]. However, it did not address the association with T2D. Although participants devoid of T2D had a higher MD2 than participants with T2D with regard to bivariate analysis, the differences were no longer statistically significant after multivariable adjustment. This result may be explained by two reasons: the Sofi score may not be adapted to the Swiss population or that adherence to this score is not associated with T2D but only with other CVD risk factors. To estimate its clinical interest, further studies are needed to assess the prospective association between this score and incident T2D.

### 4.3. Implications for Practice and Future Research

Several studies have shown that people following a MD tend to reduce their risk of CVD, stroke, obesity, T2D, hypertension, several types of cancer, allergic diseases, and degenerative diseases such as Alzheimer’s and Parkinson’s [35,36]. Therefore, and despite our negative findings, a healthy diet has a very important role in the prevention of T2D [37] and should always be recommended to patients at risk of T2D or already presenting with T2D [38].

Future research should focus on the prospective associations between MD and incident T2D in non-Mediterranean countries, and on finding the best method to compute adherence to MD.

### 4.4. Strengths and Limitations

This study was based on a population-based sample and used a validated FFQ to assess dietary intake. It used several definitions of T2D and of the Mediterranean diet and multiple surveys to consolidate the findings.

This study also has several limitations. Firstly, it was conducted in a single location in Switzerland, and it is possible that findings might not be replicated in other parts of the country as dietary intake varies according to region [17]. Secondly, dietary intake was self-reported, and it is possible that a reporting bias might have occurred. Still, a previous study showed that participants with diagnosed T2D had a dietary intake of lesser quality than participants devoid of T2D, although the differences were relatively small [30]. Hence, it is possible that the dietary differences reported between participants with and without T2D were too small to reach statistical significance.

## 5. Conclusions

We found no association between adherence to the Mediterranean diet and T2D in a non-Mediterranean population.

## Figures and Tables

**Table 1:** Bivariate and multivariable analyses of the associations between the different Mediterranean diet scores and type 2 diabetes as defined by fasting plasma glucose ≥7 mmol/L, stratified by survey period, CoLaus|PsyCoLaus study, Lausanne, Switzerland.

		First FU			Second FU			Third FU	
	No T2D	T2D	*p*-Value	No T2D	T2D	*p*-Value	No T2D	T2D	*p*-Value
Sample Size	3967	431		2871	249		2170	197	
**Mediterranean diet 1**									
Score	4.0 ± 1.5	3.9 ± 1.5	0.339	4.0 ± 1.5	3.8 ± 1.6	0.130	4.0 ± 1.5	3.6 ± 1.4	0.002
Score §	3.97 ± 0.02	3.93 ± 0.07	0.583	3.98 ± 0.03	4.08 ± 0.10	0.325	3.97 ± 0.03	3.83 ± 0.11	0.216
Adherence (%)			0.141			0.142			0.001
Low (0–3)	1522 (38.4)	186 (43.2)		1071 (37.3)	107 (43.0)		814 (37.5)	99 (50.3)	
Medium (4–5)	1807 (45.6)	178 (41.3)		1305 (45.5)	108 (43.4)		975 (44.9)	76 (38.6)	
High (6–9)	638 (16.1)	67 (15.6)		495 (17.2)	34 (13.7)		381 (17.6)	22 (11.2)	
Adherence §									
Low (0–3)	1 (ref)			1 (ref)			1 (ref)		
Medium (4–5)	0.87 (0.68–1.10)		0.248	1.04 (0.76–1.42)		0.814	0.73 (0.53–1.03)		0.072
High (6–9)	0.89 (0.64–1.24)		0.480	1.07 (0.68–1.67)		0.773	0.61 (0.37–1.02)		0.061
**Mediterranean diet 2**									
Score	6.0 ± 2.1	5.7 ± 2.2	0.002	6.1 ± 2.1	5.9 ± 2.1	0.067	6.1 ± 2.1	5.7 ± 1.9	0.007
Score §	6.00 ± 0.03	5.95 ± 0.10	0.612	6.10 ± 0.04	6.16 ± 0.13	0.633	6.10 ± 0.04	5.93 ± 0.14	0.263
Adherence (%)			0.006			0.556			0.018
Low (0–4)	973 (24.5)	136 (31.6)		663 (23.1)	59 (23.7)		503 (23.2)	56 (28.4)	
Medium (5–8)	2481 (62.5)	246 (57.1)		1840 (64.1)	164 (65.9)		1385 (63.9)	128 (65.0)	
High (9–14)	513 (12.9)	49 (11.4)		368 (12.8)	26 (10.4)		281 (13.0)	13 (6.6)	
Adherence §									
Low (0–4)	1 (ref)			1 (ref)			1 (ref)		
Medium (5-8)	0.90 (0.70–1.15)		0.389	1.16 (0.82–1.63)		0.410	0.93 (0.65–1.34)		0.714
High (9–14)	1.03 (0.69–1.53)		0.896	1.40 (0.81–2.41)		0.229	0.55 (0.28–1.08)		0.082

FU, follow-up. Results are expressed as the number of participants (column percentage) or as odds ratio and (95% confidence interval) for categorical variables and as mean ± standard deviation (bivariate) or adjusted mean ± standard error (multivariable, §) for continuous variables. Between-group comparisons were performed using chi-square (bivariate) or logistic regression (multivariable) for categorical variables and analysis of variance for continuous variables. § adjusted on age (continuous), sex, place of birth (Swiss-born, yes or no), educational level (high, medium, low), smoking categories (never, former, current), BMI categories (normal, overweight, obese), hypertension (yes or no), alcohol consumption (yes or no), and total caloric intake (continuous).

**Table 2:** Bivariate and multivariable analyses of the associations between the different Mediterranean diet scores and type 2 diabetes as defined by glycated hemoglobin ≥6.5% (48 mmol/mol), stratified by survey period, CoLaus|PsyCoLaus study, Lausanne, Switzerland.

		Second FU			Third FU	
	No T2D	T2D	*p*-Value	No T2D	T2D	*p*-Value
Sample Size	2881	236		2188	174	
**Mediterranean diet 1**						
Score	4.0 ± 1.5	3.8 ± 1.5	0.033	4.0 ± 1.5	3.6 ± 1.4	0.001
Score §	3.98 ± 0.03	4.02 ± 0.10	0.692	3.97 ± 0.03	3.80 ± 0.11	0.150
Adherence (%)			0.028			<0.001
Low (0–3)	1074 (37.3)	103 (43.6)		820 (37.5)	91 (52.3)	
Medium (4–5)	1306 (45.3)	106 (44.9)		984 (45.0)	64 (36.8)	
High (6–9)	501 (17.4)	27 (11.4)		384 (17.6)	19 (10.9)	
Adherence §						
Low (0–3)	1 (ref)			1 (ref)		
Medium (4–5)	1.06 (0.77–1.46)		0.702	0.68 (0.47–0.97)		0.032
High (6–9)	0.88 (0.54–1.43)		0.614	0.59 (0.35–1.02)		0.060
**Mediterranean diet 2**						
Score	6.1 ± 2.1	5.8 ± 2.1	0.041	6.1 ± 2.1	5.8 ± 2.0	0.054
Score §	6.10 ± 0.04	6.09 ± 0.13	0.900	6.09 ± 0.04	6.00 ± 0.15	0.578
Adherence (%)			0.485			0.289
Low (0–4)	664 (23.1)	58 (24.6)		512 (23.4)	47 (27.0)	
Medium (5–8)	1848 (64.1)	154 (65.3)		1397 (63.9)	111 (63.8)	
High (9–14)	369 (12.8)	24 (10.2)		278 (12.7)	16 (9.2)	
Adherence §						
Low (0–4)	1 (ref)			1 (ref)		
Medium (5-8)	1.06 (0.75–1.50)		0.751	0.98 (0.67–1.44)		0.918
High (9–14)	1.20 (0.68–2.11)		0.525	0.85 (0.45–1.63)		0.630

FU, follow-up. Results are expressed as the number of participants (column percentage) or as odds ratio and (95% confidence interval) for categorical variables and as mean ± standard deviation (bivariate) or adjusted mean ± standard error (multivariable, §) for continuous variables. Between-group comparisons were performed using chi-square (bivariate) or logistic regression (multivariable) for categorical variables and analysis of variance for continuous variables. § adjusted on age (continuous), sex, place of birth (Swiss-born, yes or no), educational level (high, medium, low), smoking categories (never, former, current), BMI categories (normal, overweight, obese), hypertension (yes or no), alcohol consumption (yes or no), and total caloric intake (continuous).

## Data Availability

The data of CoLaus|PsyCoLaus study used in this article cannot be fully shared as they contain potentially sensitive personal information on participants. According to the Ethics Committee for Research of the Canton of Vaud, sharing these data would be a violation of the Swiss legislation with respect to privacy protection. However, coded individual-level data that do not allow researchers to identify participants are available upon request to researchers who meet the criteria for data sharing of the CoLaus|PsyCoLaus Datacenter (CHUV, Lausanne, Switzerland). Any researcher affiliated to a public or private research institution who complies with the CoLaus|PsyCoLaus standards can submit a research application to research.colaus@chuv.ch or research.psycolaus@chuv.ch. Proposals requiring baseline data only, will be evaluated by the baseline (local) Scientific Committee (SC) of the CoLaus and PsyCoLaus studies. Proposals requiring follow-up data will be evaluated by the follow-up (multicentric) SC of the CoLaus|PsyCoLaus cohort study. Detailed instructions for gaining access to the CoLaus|PsyCoLaus data used in this study are available at www.colaus-psycolaus.ch/professionals/how-to-collaborate/ accessed on 9 June 2023.

## Supplementary Materials

The following supporting information can be downloaded at: https://www.mdpi.com/article/10.3390/nu15133025/s1, Table S1: Items used for computation of Sofi score; Table S2: Characteristics of included and excluded participants for each follow-up, CoLaus|PsyColaus study, Lausanne, Switzerland; Table S3: Characteristics of the participants according to categories of the Mediterranean diet (definition 1), for each follow-up, CoLaus|PsyColaus study, Lausanne, Switzerland; Table S4: Characteristics of the participants according to categories of the Mediterranean diet (as per Sofi score), for each follow-up, CoLaus|PsyColaus study, Lausanne, Switzerland; Table S5: Multivariable analysis of the associations between the different Mediterranean diet scores and type 2 diabetes as defined by fasting plasma glucose ≥ 7 mmol/L or glycated haemoglobin ≥ 6.5% (48 mmol/mol), stratified by survey period and weighted for non-inclusion, CoLaus|PsyCoLaus study, Lausanne, Switzerland; Table S6: Bivariate and multivariable analysis of fat consumption of participants with and without diabetes for each follow-up, CoLaus|PsyColaus study, Lausanne, Switzerland.
