# Supplementary material for: Association between Mediterranean Diet and Type 2 Diabetes: Multiple Cross-Sectional Analyses
Source: Nutrients. 2023 Jul 3;15(13):3025. doi: 10.3390/nu15133025 (PMC10346215; doi:10.3390/nu15133025)
Supplement: Supplementary file 1 [file nutrients-15-03025-s001.zip › nutrients-2471538-supplementary.pdf]

Supplementary information

Table S1: Items used for computation of Sofi score.

Score component	Items from the food frequency questionnaire
Fruit	Banana, apple, pear, plum, grapes, gr/day
	Citrus fruits (orange, mandarin), gr/day
	Peach, apricot, melon, gr/day
	Berries (strawberries, blueberries), gr/day
	Kiwi, gr/day
	Fruit, preserve, gr/day
	Fresh fruit juice, mL/day
	Bottled fruit juice, mL/day
Vegetables	Green beans, spinach, gr/day
	Cauliflower, broccoli, gr/day
	Tomatoes, gr/day
	Carrots, gr/day
	Green salad, gr/day
	Green peas, corn, maize, gr/day
	Vegetable broth, gr/day
	Vegetable soup (peas, beans, minestrone), gr/day
Cereals	Whole wheat bread, rye bread, gr/day
	Müesli, gr/day
	Corn-flakes, puffed rice, gr/day
	Rusks, Swedish bread, gr/day
	Rice, gr/day
	Couscous, semolina, gr/day
Fish	Salmon (fresh or smoked), gr/day
	Fried or breaded fish, gr/day
	Tuna in oil, gr/day
	White fish (cod, trout, hake), gr/day
Meat	Beef, horse, veal, gr/day
	Chicken, skinned, gr/day
	Chicken, with skin, gr/day
	Hamburger, rib steak, roasted meat (Beef, horse or veal),
	Cured ham, mutton or pork chops, gr/day
	Sausage, salami, ham, gr/day
	Pâté, terrine, gr/day
	Cervelas, wieners, gr/day
	Frankfurter, small sausages, gr/day
Dairy	Plain yoghurt, gr/day
	Low-fat yoghurt, gr/day
	Fruit/aroma yoghurt, gr/day
	Cottage cheese (0% fat), gr/day
	Cottage cheese, ricotta, gr/day
	Feta, mozzarella, gr/day
	Gruyère, tomme, camembert, gr/day
	Cheese fondue, gr/day
	Milk as drink (0% fat), mL/day
	Milk as drink (non 0% fat), mL/day
Alcohol	Alcohol consumption in gr/day
Olive oil	Olive oil (for cooking), gr/day

All fruits and all vegetables, including canned and juice. https://www.sge-ssn.ch/fr/toi-et-moi/boire-et-manger/equilibre-alimentaire/pyramide-alimentaire-suisse/

Table S2: characteristics of included and excluded participants for each follow-up, CoLaus|PsyColaus study, Lausanne, Switzerland.

	First follow-up (2009-2012)			Second follow-up (2014-2017)			Third follow-up (2018-2021)		
	**Included**	**Excluded**	**P-value**	**Included**	**Excluded**	**P-value**	**Included**	**Excluded**	**P-value**
	**4398**	**Varies**		**3154**	**1727**		**2394**	**1355**	
Men (%)	2008 (45.7)	349 (52.4)	0.001	1418 (45.0)	774 (44.8)	0.924	1082 (45.2)	585 (43.1)	0.217
Age (years)	57.7 ± 10.5	58.5 ± 10.9	0.071	61.7 ± 9.9	65.2 ± 11.1	<0.001	65.0 ± 9.6	65.8 ± 10.4	0.010
Swiss born (%)	2866 (65.2)	318 (47.8)	<0.001	2112 (67.0)	950 (55.0)	<0.001	1618 (67.6)	759 (55.9)	<0.001
Educational level (%)			<0.001			<0.001			<0.001
High	971 (22.1)	108 (16.3)		763 (24.2)	268 (15.6)		565 (23.6)	281 (20.7)	
Medium	1170 (26.6)	136 (20.5)		895 (28.4)	363 (21.1)		691 (28.9)	334 (24.6)	
Low	2255 (51.3)	419 (63.2)		1495 (47.4)	1093 (63.4)		1137 (47.5)	741 (54.7)	
Body mass index (kg/m²)	26.1 ± 4.5	27.2 ± 5.3	<0.001	26.2 ± 4.6	27.0 ± 5.0	<0.001	26.2 ± 4.5	26.7 ± 5.0	<0.001
BMI categories (%)			<0.001			<0.001			0.001
Normal	1961 (44.6)	219 (36.4)		1383 (43.9)	474 (35.7)		1038 (43.4)	463 (40.1)	
Overweight	1715 (39.0)	245 (40.8)		1241 (39.4)	534 (40.2)		930 (38.9)	426 (36.9)	
Obese	722 (16.4)	137 (22.8)		530 (16.8)	319 (24.0)		426 (17.8)	266 (23.0)	
Smoking status (%)			<0.001			0.004			0.309
Never	1802 (41.0)	233 (38.3)		1335 (42.3)	551 (41.1)		1058 (44.2)	437 (43.7)	
Former	1682 (38.2)	201 (33.0)		1258 (39.9)	496 (37.0)		967 (40.4)	389 (38.9)	
Current	914 (20.8)	175 (28.7)		561 (17.8)	294 (21.9)		369 (15.4)	175 (17.5)	
Hypertension (%)	1788 (40.7)	308 (47.0)	0.002	1369 (43.4)	894 (58.4)	<0.001	1175 (49.1)	686 (55.3)	<0.001
Alcohol consumption (%)	3368 (76.6)	415 (62.3)	<0.001	2420 (76.7)	645 (59.4)	<0.001	1748 (73)	311 (22.9)	<0.001
Diabetes (%)	431 (9.8)	108 (16.7)	<0.001	256 (8.1)	242 (17.3)	<0.001	202 (8.4)	181 (15.5)	<0.001

Results are expressed as number of participants (column percentage) for categorical variables and as mean ± standard deviation for continuous variables. Between-group comparisons performed using chi-square for categorical variables and student's t-test for continuous variables.

Table S3: characteristics of the participants according to categories of the Mediterranean diet (definition 1), for each follow-up, CoLaus|PsyColaus study, Lausanne, Switzerland.

	**First follow-up (2009-2012)**				**Second follow-up (2014-2017)**			
	**Low (0-3)**	**Medium (4-5)**	**High (6-9)**	**P-value**	**Low (0-3)**	**Medium (4-5)**	**High (6-9)**	**P-value**
**Sample size**	**1706**	**1985**	**705**		**1178**	**1412**	**529**	
Men (%)	747 (43.7)	903 (45.5)	358 (50.8)	0.007	519 (44.1)	624 (44.2)	265 (50.1)	0.042
Age (years)	57.7 ± 10.6	57.5 ± 10.4	58.1 ± 10.1	0.325	61.7 ± 9.9	62.1 ± 10.1	60.5 ± 9.0	0.006
Swiss born (%)	1204 (70.5)	1267 (63.8)	395 (56.0)	<0.001	857 (72.8)	922 (65.3)	316 (59.7)	<0.001
Educational level (%)				<0.001				<0.001
High	312 (18.3)	472 (23.8)	187 (26.5)		222 (18.9)	367 (26.0)	170 (32.1)	
Medium	442 (25.9)	526 (26.5)	202 (28.7)		310 (26.3)	418 (29.6)	159 (30.1)	
Low	952 (55.8)	987 (49.7)	316 (44.8)		646 (54.8)	627 (44.4)	200 (37.8)	
Body mass index (kg/m^2)	26.5 ± 4.7	25.8 ± 4.4	25.7 ± 4.2	<0.001	26.8 ± 4.8	25.9 ± 4.4	25.5 ± 4.3	<0.001
BMI categories (%)				<0.001				<0.001
Normal	705 (41.3)	936 (47.2)	320 (45.4)		452 (38.4)	642 (45.4)	274 (51.8)	
Overweight	677 (39.6)	751 (37.8)	287 (40.7)		486 (41.3)	564 (39.9)	179 (33.8)	
Obese	326 (19.1)	298 (15.0)	98 (13.9)		240 (20.4)	207 (14.7)	76 (14.4)	
Smoking status (%)				<0.001				<0.001
Never	708 (41.5)	805 (40.6)	289 (41.0)		528 (44.8)	578 (40.9)	214 (40.5)	
Former	596 (34.9)	783 (39.5)	303 (43.0)		412 (35.0)	603 (42.7)	232 (43.9)	
Current	404 (23.7)	397 (20.0)	113 (16.0)		238 (20.2)	232 (16.4)	83 (15.7)	
Total energy intake (kcal)	1516 [1202 - 1879]	1673 [1329 - 2120]	1852 [1472 - 2205]	§ <0.001	1513 [1194 - 1857]	1680 [1343 - 2087]	1799 [1481 - 2147]	§ <0.001
Hypertension (%)	715 (41.9)	772 (38.9)	301 (42.7)	0.090	546 (46.4)	616 (43.6)	188 (35.5)	<0.001
Alcohol consumption (%)	1203 (70.4)	1548 (78.0)	617 (87.5)	<0.001	860 (73.0)	1084 (76.7)	459 (86.8)	<0.001
Family history of diabetes (%)	379 (22.2)	431 (21.7)	144 (20.4)	0.633	273 (23.2)	296 (21.0)	115 (21.7)	0.392

Results are expressed as number of participants (column percentage) for categorical variables and as mean ± standard deviation or median [interquartile range] for continuous variables. Between-group comparisons performed using chi-square for categorical variables and analysis of variance or Kruskal-Wallis nonparametric test (§) for continuous variables.

Table S3 (continued): characteristics of the participants according to categories of the Mediterranean diet (definition 1), for each follow-up, CoLaus|PsyColaus study, Lausanne, Switzerland.

	Third follow-up (2018-2021)			
	**Low (0-3)**	**Medium (4-5)**	**High (6-9)**	**P-value**
**Sample size**	**913**	**1050**	**403**	
Men (%)	400 (43.8)	469 (44.6)	201 (49.9)	0.110
Age (years)	65.1 ± 9.8	64.7 ± 9.4	64.8 ± 9.3	0.612
Swiss born (%)	663 (72.6)	685 (65.2)	252 (62.5)	<0.001
Educational level (%)				<0.001
High	179 (19.6)	254 (24.2)	130 (32.3)	
Medium	251 (27.5)	309 (29.4)	124 (30.8)	
Low	483 (52.9)	487 (46.4)	149 (37.0)	
Body mass index (kg/m^2)	26.7 ± 4.5	25.8 ± 4.5	25.6 ± 4.3	<0.001
BMI categories (%)				<0.001
Normal	346 (37.9)	493 (46.9)	190 (47.2)	
Overweight	371 (40.6)	391 (37.2)	157 (39.0)	
Obese	196 (21.5)	167 (15.9)	56 (13.9)	
Smoking status (%)				0.005
Never	391 (42.8)	482 (45.9)	174 (43.2)	
Former	350 (38.3)	432 (41.1)	175 (43.4)	
Current	172 (18.8)	137 (13.0)	54 (13.4)	
Total energy intake (kcal)	1429 [1144 - 1781]	1565 [1240 - 1954]	1704 [1403 - 2102]	§ <0.001
Hypertension (%)	472 (51.7)	501 (47.7)	185 (45.9)	0.084
Alcohol consumption (%)	617 (67.6)	777 (73.9)	344 (85.4)	<0.001
Family history of diabetes (%)	218 (23.9)	241 (22.9)	85 (21.1)	0.541

Results are expressed as number of participants (column percentage) for categorical variables and as mean ± standard deviation or median [interquartile range] for continuous variables. Between-group comparisons performed using chi-square for categorical variables and analysis of variance or Kruskal-Wallis nonparametric test (§) for continuous variables.

Table S4: characteristics of the participants according to categories of the Mediterranean diet (as per Sofi score), for each follow-up, CoLaus|PsyColaus study, Lausanne, Switzerland.

	**First follow-up (2009-2012)**				**Second follow-up (2014-2017)**			
	**Low (0-4)**	**Medium (5-8)**	**High (9-14)**	**P-value**	**Low (0-4)**	**Medium (5-8)**	**High (9-14)**	**P-value**
**Sample size**	**1109**	**2727**	**562**		**722**	**2003**	**394**	
Men (%)	659 (59.4)	1191 (43.7)	158 (28.1)	<0.001	395 (54.7)	897 (44.8)	116 (29.4)	<0.001
Age (years)	57.5 ± 10.5	57.7 ± 10.5	58.1 ± 10.2	0.529	61.1 ± 9.5	62.0 ± 10.0	61.4 ± 9.7	0.110
Swiss born (%)	800 (72.1)	1760 (64.5)	306 (54.5)	<0.001	542 (75.1)	1338 (66.8)	215 (54.6)	<0.001
Educational level (%)				0.002				0.002
High	205 (18.5)	633 (23.2)	133 (23.7)		156 (21.6)	494 (24.7)	109 (27.7)	
Medium	282 (25.5)	729 (26.7)	159 (28.3)		189 (26.2)	568 (28.4)	130 (33.0)	
Low	621 (56.1)	1364 (50.0)	270 (48.0)		377 (52.2)	941 (47.0)	155 (39.3)	
Body mass index (kg/m^2)	26.4 ± 4.4	25.9 ± 4.4	25.9 ± 4.9	0.009	26.7 ± 4.7	26.1 ± 4.4	25.4 ± 5	<0.001
BMI categories (%)				0.005				<0.001
Normal	451 (40.7)	1235 (45.3)	275 (48.9)		276 (38.2)	877 (43.8)	215 (54.6)	
Overweight	451 (40.7)	1069 (39.2)	195 (34.7)		313 (43.4)	801 (40.0)	115 (29.2)	
Obese	207 (18.7)	423 (15.5)	92 (16.4)		133 (18.4)	326 (16.3)	64 (16.2)	
Smoking status (%)				<0.001				0.001
Never	359 (32.4)	1154 (42.3)	289 (51.4)		282 (39.1)	848 (42.3)	190 (48.2)	
Former	454 (40.9)	1036 (38.0)	192 (34.2)		280 (38.8)	822 (41.0)	145 (36.8)	
Current	296 (26.7)	537 (19.7)	81 (14.4)		160 (22.2)	334 (16.7)	59 (15.0)	
Total energy intake (kcal)	1488	1652	1895	§ <0.001	1486	1645	1866	§ <0.001
	[1175 - 1863]	[1304 - 2069]	[1546 - 2265]		[1166 - 1838]	[1315 - 2024]	[1521 - 2276]	
Hypertension (%)	492 (44.4)	1075 (39.4)	221 (39.3)	0.015	316 (43.8)	897 (44.8)	137 (34.8)	0.001
Alcohol consumption (%)	915 (82.5)	2089 (76.6)	364 (64.8)	<0.001	607 (84.1)	1528 (76.3)	268 (68.0)	<0.001
Family history of diabetes (%)	234 (21.1)	595 (21.8)	125 (22.2)	0.838	157 (21.8)	452 (22.6)	75 (19.0)	0.301

Results are expressed as number of participants (column percentage) for categorical variables and as mean ± standard deviation or median [interquartile range] for continuous variables. Between-group comparisons performed using chi-square for categorical variables and analysis of variance or Kruskal-Wallis nonparametric test (§) for continuous variables.

Table S4 (continued): characteristics of the participants according to categories of the Mediterranean diet (as per Sofi score), for each follow-up, CoLaus|PsyColaus study, Lausanne, Switzerland.

	Third follow-up (2018-2021)			
	**Low (0-4)**	**Medium (5-8)**	**High (9-14)**	**P-value**
**Sample size**	**559**	**1512**	**294**	
Men (%)	305 (54.6)	677 (44.8)	88 (29.9)	<0.001
Age (years)	64.6 ± 9.6	64.9 ± 9.7	65.1 ± 8.8	0.690
Swiss born (%)	408 (73.0)	1031 (68.1)	160 (54.4)	<0.001
Educational level (%)				0.015
High	109 (19.5)	382 (25.3)	72 (24.5)	
Medium	154 (27.6)	435 (28.8)	94 (32.0)	
Low	296 (53.0)	695 (46.0)	128 (43.5)	
Body mass index (kg/m^2)	26.4 ± 4.3	26.1 ± 4.5	25.6 ± 4.8	0.043
BMI categories (%)				0.165
Normal	227 (40.6)	657 (43.4)	144 (49.0)	
Overweight	221 (39.5)	595 (39.3)	103 (35.0)	
Obese	111 (19.9)	261 (17.3)	47 (16.0)	
Smoking status (%)				<0.001
Never	220 (39.4)	673 (44.5)	153 (52.0)	
Former	221 (39.5)	623 (41.2)	113 (38.4)	
Current	118 (21.1)	217 (14.3)	28 (9.5)	
Total energy intake (kcal)	1380 [1119 - 1707]	1561 [1254 - 1925]	1778 [1433 - 2242]	§ <0.001
Hypertension (%)	303 (54.2)	723 (47.8)	132 (44.9)	0.012
Alcohol consumption (%)	457 (81.8)	1089 (72.0)	191 (65.0)	<0.001
Family history of diabetes (%)	121 (21.7)	359 (23.7)	64 (21.8)	0.526

Results are expressed as number of participants (column percentage) for categorical variables and as mean ± standard deviation or median [interquartile range] for continuous variables. Between-group comparisons performed using chi-square for categorical variables and analysis of variance or Kruskal-Wallis nonparametric test (§) for continuous variables.

Table S5. Multivariable analysis of the associations between the different Mediterranean diet scores and type 2 diabetes as defined by fasting plasma glucose ≥7 mmol/L or glycated haemoglobin ≥6.5% (48 mmol/mol), stratified by survey period and weighted for non-inclusion, CoLaus|PsyCoLaus study, Lausanne, Switzerland.

	First follow-up	P-value	Second follow-up	P-value	Third follow-up	P-value
**Diabetes by FPG**						
**Mediterranean diet 1**						
Adherence §						
Low (0-3)	1 (ref)		1 (ref)		1 (ref)	
Medium (4-5)	0.87 (0.68 - 1.11)	0.265	1.05 (0.76 - 1.44)	0.772	0.73 (0.51 - 1.04)	0.077
High (6-9)	0.90 (0.64 - 1.26)	0.540	1.09 (0.69 - 1.73)	0.703	0.69 (0.41 - 1.15)	0.155
**Mediterranean diet 2**						
Adherence §						
Low (0-4)	1 (ref)		1 (ref)		1 (ref)	
Medium (5-8)	0.90 (0.70 - 1.17)	0.439	1.20 (0.85 - 1.69)	0.310	0.9 (0.61 - 1.31)	0.570
High (9-14)	1.05 (0.70 - 1.58)	0.816	1.46 (0.86 - 2.48)	0.166	0.56 (0.28 - 1.10)	0.091
**Diabetes by glycated haemoglobin**						
**Mediterranean diet 1**						
Adherence §						
Low (0-3)	NA		1 (ref)		1 (ref)	
Medium (4-5)	NA		1.09 (0.79 - 1.51)	0.613	0.68 (0.46 - 0.99)	0.042
High (6-9)	NA		0.92 (0.56 - 1.52)	0.756	0.68 (0.39 - 1.19)	0.175
**Mediterranean diet 2**						
Adherence §						
Low (0-4)	NA		1 (ref)		1 (ref)	
Medium (5-8)	NA		1.09 (0.76 - 1.56)	0.635	0.92 (0.61 - 1.39)	0.691
High (9-14)	NA		1.25 (0.72 - 2.19)	0.429	0.83 (0.43 - 1.60)	0.574

FPG, fasting plasma glucose; NA, not available. Results are expressed as odds ratio and (95% confidence interval), adjusted on age (continuous), sex, Swiss born (yes, no), educational level (high, medium, low), smoking categories (never, former, current), BMI categories (normal, overweight, obese), hypertension (yes, no), alcohol consumption (yes, no) and total caloric intake (continuous).

Table S6: bivariate and multivariable analysis of fat consumption of participants with and without diabetes for each follow-up, CoLaus|PsyColaus study, Lausanne, Switzerland.

	First follow-up (2009-2012)			Second follow-up (2014-2017)			Third follow-up (2018-2021)		
	**No diabetes**	**Diabetes**	**P-value**	**No diabetes**	**Diabetes**	**P-value**	**No diabetes**	**Diabetes**	**P-value**
	**3967**	**431**		**2895**	**256**		**2192**	**202**	
**Bivariate**									
Types of fat (kcal/day)									
Total	584 [445-763]	610 [439-795]	0.099	608 [465-772]	631 [480-802]	0.157	643 [501-829]	616 [485-789]	0.161
Saturated	213 [156-290]	223 [162-305]	0.055	216 [161-295]	234 [167-308]	0.043	238 [178-313]	239 [175-302]	0.623
Monounsaturated	232 [174-305]	241 [170-310]	0.410	244 [185-316]	243 [185-326]	0.820	261 [201-339]	244 [183-328]	0.044
Polyunsaturated	81 [60-107]	85 [62-114]	0.014	81 [61-106]	87 [62-118]	0.041	86 [66-111]	79 [62-107]	0.126
**Multivariable**									
Types of fat (kcal/day)									
Total	624 ± 2	631 ± 7	0.287	640 ± 3	647 ± 9	0.474	683 ± 3	678 ± 10	0.630
Saturated	234 ± 1	233 ± 3	0.794	237 ± 1	235 ± 4	0.638	256 ± 1	253 ± 5	0.641
Monounsaturated	248 ± 1	253 ± 3	0.126	261 ± 1	265 ± 5	0.410	279 ± 2	277 ± 5	0.745
Polyunsaturated	88 ± 1	91 ± 2	0.076	88 ± 1	93 ± 2	0.010	92 ± 1	92 ± 2	0.689

Results are expressed as median [interquartile range] for bivariate analysis and as adjusted mean ± standard error for multivariable analysis. Between-group comparisons performed using Kruskal-Wallis test (bivariate) and analysis of variance (multivariable). Multivariable analysis adjusted on age (continuous), sex, Swiss born (yes, no), educational level (high, medium, low), smoking categories (never, former, current), BMI categories (normal, overweight, obese), hypertension (yes, no), alcohol consumption (yes, no) and total caloric intake (continuous).
